# Multidrug Resistance and Virulence Gene Profiles of *E. coli* in Broiler Chickens: A Study From Noakhali, Bangladesh

**DOI:** 10.1155/vmi/1157843

**Published:** 2025-11-25

**Authors:** Mohammad Sharif Uddin, Md. Habib Ullah Masum, Md. Razib Hosen, Suhag Chandra Roy, A. B. Z. Naimur Rahman, Noimul Hasan Siddiquee, Afifa Siddiqua, Imam Hossain, Tania Peas

**Affiliations:** ^1^Department of Microbiology, Noakhali Science and Technology University (NSTU), Noakhali 3814, Bangladesh; ^2^Department of Genomics and Bioinformatics, Chattogram Veterinary and Animal Sciences University (CVASU), Khulshi, Chattogram 4225, Bangladesh; ^3^Bioinformatics Division, National Institute of Biotechnology, Ashulia, Savar, Dhaka 1349, Bangladesh; ^4^Department of Physiology, Biochemistry and Pharmacology, Chattogram Veterinary and Animal Sciences University (CVASU), Khulshi, Chattogram 4225, Bangladesh

**Keywords:** antibiotic resistance, APEC, Bangladesh, EEC, multidrug resistance, Noakhali, poultry, virulence gene

## Abstract

Avian pathogenic *Escherichia coli* (APEC) is the primary cause of colibacillosis, a significant bacterial disease in poultry associated with high mortality rates and substantial economic losses. In Bangladesh, the poultry sector is crucial in ensuring food security and supporting livelihoods, yet APEC poses a significant challenge. The extensive use of antibiotics has heightened antimicrobial resistance (AMR), undermining the efficacy of therapeutic alternatives and raising public health issues. The present study assessed the prevalence, AMR, and virulence gene profiles of APEC and their environs (environmental *E. coli* [EEC]) in Bangladesh. All isolates demonstrated significant resistance, with more than 90% resistant to the most frequently used antibiotics (tetracycline, ciprofloxacin, ampicillin, and levofloxacin). The EEC isolates demonstrated a notably higher level of resistance compared to APEC (*p* < 0.05), indicating a greater exposure to antimicrobials in the environment. The prevalence of multidrug resistance (MDR) was also notably high (98.94%). The study also profiled virulence-associated genes (VAGs), with the iron acquisition gene *iro*N being the most prevalent (69.5%), followed by *omp*T (58.8%) and *hly*F (53.7%), indicating strong pathogenic potential in both APEC and EEC. However, the VAGs' distribution showed no significant difference between APEC and EEC, suggesting possible environmental reservoirs for the pathogenic strains. The existence of multiple VAGs, along with elevated resistance levels, emphasizes the dual threat posed by these isolates to both poultry and public health. Overall, the findings underscore the urgent need for improved biosecurity practices, prudent antibiotic use, and ongoing surveillance to mitigate the risks posed by resistant and virulent bacterial strains.

## 1. Introduction

*Escherichia coli (E. coli)*, a Gram-negative and nonspore-forming bacterium, is widely distributed and typically prevails as the predominant species in the gut microbiota of humans, animals, and birds [1, 2]. Pathogenic *E. coli* strains exist alongside commensal counterparts, posing a significant threat by infecting the intestines and other organs. Notably, avian pathogenic *E. coli* (APEC) is a major concern in poultry, as it is responsible for causing extraintestinal infections that can severely impact bird health and productivity [2, 3]. Colibacillosis, marked by both localized and systemic infections, is the consequence of the infection of APEC in various avian species. The condition impacts commercial poultry, including chickens, ducks, turkeys, and other avian species, posing substantial health and economic issues to the poultry industry [2, 4]. Colibacillosis manifests in several clinical forms, ranging from acute septicemia to more subacute conditions. These can include pericarditis, airsacculitis, arthritis, perihepatitis, peritonitis, and salpingitis, which often progress to septicemia and, in severe cases, result in death [2, 5]. Several studies have reported that colibacillosis can lead to significant mortality in poultry, with death rates reaching up to 20%. In addition to mortality, the disease contributes to considerable morbidity, negatively impacting poultry productivity. Affected flocks experience an approximate 2% reduction in live body weight and a 2.7% decline in feed conversion efficiency, which have serious economic implications for the poultry industry [2, 3]. Furthermore, poultry suffering from colibacillosis can experience a reduction in egg production by as much as 20%, along with decreased hatchability rates. Additionally, the condition significantly increases the likelihood of carcass condemnation at slaughter, with rates potentially reaching up to 43% [2, 6].

APEC strains are known for their diverse array of virulence factors. These include components such as lipopolysaccharide and capsule complexes, as well as adhesins, toxins, colicin plasmids, iron acquisition systems, F hemolysin, serum resistance proteins, and temperature-sensitive hemagglutinin [2, 3, 7, 8]. The major virulence-associated genes (VAGs) involved in colibacillosis are *iut*A, *hly*F, *iss*, *iro*N, *omp*T, *fim*H, *cvi/cva*, *iuc*D, *tra*T, *kps*MTII, *tsh*, and *aer*J, as they are mostly present in APEC [7–10]. These genes are encoded on a pathogenicity island (PAI), plasmid, and other mobile elements. The simultaneous presence of antimicrobial resistance (AMR) genes and virulence genes, often located on plasmids, significantly increases the likelihood of horizontal gene transfer between bacterial populations [2, 11]. Certain strains of *E. coli* can obtain novel genes via horizontal gene transfer, enhancing their virulence and enabling a broader spectrum of infections in both animals and humans [12, 13]. APEC may serve as a reservoir for virulence and AMR genes, which could be transferred to extraintestinal pathogenic *E. coli* (ExPEC) strains in humans. This suggests a potential zoonotic transmission pathway, where genetic elements from APEC in poultry could contribute to the emergence of drug-resistant or more virulent ExPEC strains in humans [2]. However, human ExPECs and APEC have avian-associated ColV plasmids that share 10 major virulence genes (*iuc*C, *iuc*D, *iut*A, *cva*A, *ets*A, *hly*F, *omp*T, *cva*B, *cva*C, and *cvi*) [7, 9, 14]. Transmission of these strains to human beings might happen by direct contact with animals, exposure to animal excrement, ingestion of undercooked meat, and contact with meat surfaces, constituting a significant public health hazard [15].

Colibacillosis is one of the most economically significant diseases in the poultry industry worldwide, including in Bangladesh. The poultry industry in Bangladesh has seen rapid growth over the past few decades, contributing significantly to the country's GDP and rural employment [16]. However, this expansion has been accompanied by the increasing prevalence of infectious diseases, with colibacillosis being one of the most prominent challenges [17]. In Bangladesh, outbreaks of colibacillosis are common, especially during the hot and humid months when environmental stress weakens the birds' immune systems, making them more susceptible to infections [4, 18]. The widespread use of antibiotics in both therapeutic and growth-promoting capacities has also led to the emergence of antibiotic-resistant strains of APEC and environmental *E. coli* (EEC), which pose a serious public health risk due to the potential for zoonotic transmission [4, 19]. Given the impact of colibacillosis on poultry production and the growing threat of antibiotic resistance, understanding the epidemiology and resistance trends of APEC isolates in the Bangladeshi poultry sector is essential. However, the study focused on the Noakhali region is limited, unlike other studies conducted in different regions of Bangladesh. This region is a coastal area with unique poultry farming practices and climatic conditions [20–25]. Data from this region have been largely undocumented. Also, this area represents a distinct ecological and management zone, characterized by unique farming practices, climatic conditions, and biosecurity standards that may influence bacterial diversity and resistance profiles. Additionally, the indiscriminate use of antimicrobials in food animals for preventive and curative purposes increases the risk of emerging and spreading antimicrobial-resistant bacterial strains [26, 27].

This study was conducted in the Noakhali region, focusing on the bacteriological and AMR patterns exhibited by APEC and EEC, as well as the molecular characterization of both strains isolated from broiler anatomical and environmental samples. The results of this research will provide unique and region-specific insights that enhance the overall comprehension of APEC epidemiology in Bangladesh and the South Asian area.

## 2. Materials and Methods

### 2.1. Sample Collection and Ethical Approval

**Figure 1:**
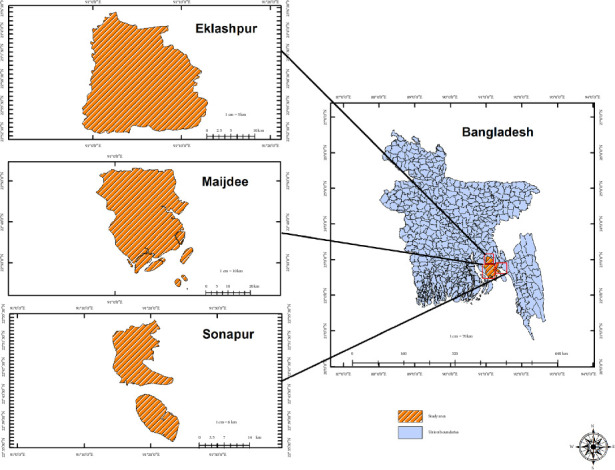
The geographical locations of the study area.

A cross-sectional study was conducted in three cities—Maijdee, Sonapur, and Eklaspur—of the Noakhali district of Bangladesh (Figure 1). The study spanned over a year, from January 2022 to February 2023. A total of 155 broiler samples were collected through a random sampling method from different locations across the city. The collection process involved obtaining samples from key anatomical sites of deceased broilers, such as the liver, lung, spleen, intestine, air sac, and feces (Supporting Figure 1). In addition to these broiler samples, environmental samples from farms, including air, feed, and water, were also collected to analyze possible contamination sources comprehensively. Each sample was carefully handled under aseptic conditions to prevent cross-contamination. The entire procedure was executed in compliance with the guidelines specified in the Guide for the Care and Use of Laboratory Animals by the National Institutes of Health. After collection, these samples were transported to the Department of Microbiology at Noakhali Science and Technology University (NSTU) for further laboratory analysis. The samples were carefully transported in an icebox kept at 4°C to ensure their integrity until they reached the laboratory.

### 2.2. Isolation and Identification of APEC and EEC

For direct inoculation, cloacal swabs collected from broilers were streaked onto MacConkey agar under sterile conditions. In the case of feed, water, and feces samples, the inoculum was first suspended in peptone water for enrichment and incubated aerobically at 37°C for 24 h. Afterward, enriched inoculum was streaked onto MacConkey agar (M081, HiMedia, India), followed by a 24-h aerobic incubation at 37°C. Air samples were directly exposed to MacConkey agar plates for an hour before incubating under the same conditions. The plates were incubated aerobically at 37°C for 24 h. After incubation, *E. coli* colonies appeared as convex-shaped, dark pink colonies on the MacConkey agar plates. A single pink colony from the primary culture was selected and subcultured onto a fresh MacConkey agar plate to ensure the purity of the culture. The pure culture was then transferred to eosin methylene blue (EMB) agar (M317, HiMedia, India), which was incubated aerobically for 24 h. On EMB agar, *E. coli* colonies exhibited a distinct metallic green sheen, characteristic of the species. These greenish colonies were identified as presumptive *E. coli* and were subsequently subcultured onto nutrient agar (HiMedia, India) for further analysis. Presumptive isolates were subjected to confirmation through a series of biochemical tests, such as the triple sugar iron (TSI) agar test and motility, indole, and urease (MIU), along with the Simmons citrate test (Supporting Table 1). The isolates were preserved at −80°C in Luria–Bertani (LB) broth (Oxoid Ltd., UK) with 30% glycerol until further use.

### 2.3. AMR Profiling

The antimicrobial susceptibility profile was performed with the Kirby–Bauer disk diffusion technique, as originally described by Bauer et al. [28]. For the AMR profiling, the following antibiotics were used: gentamicin (CN 10), ampicillin (AMP 10), nitrofurantoin (F 300), levofloxacin (LEV 5), ciprofloxacin (CIP 5), trimethoprim and sulfamethoxazole (SXT 25), and tetracyclines (TE 30) (HiMedia, India) (Table 1). These antibiotics were chosen due to their frequent usage in Bangladesh's poultry industry and their widespread availability throughout the country [19, 29–31]. The *E. coli* ATCC 25922 strain was employed as a negative control to verify the reliability of the test procedure and simultaneously served as a standard for quality control of the antibiotics assessed. The zone of complete inhibition measurement was taken in millimeters (mm) using the antibiotic zone scale. By the standards set forth by the Clinical and Laboratory Standards Institute (CLSI) (2021), classifications of resistant (R), intermediate (I), and susceptible (S) are identified using the resistance breakpoints described in Table 1 [32].

### 2.4. DNA Extraction and Quantification

The genomic DNA from the APEC and EEC isolates was extracted using a commercially available DNA extraction kit (AddPrep Genomic DNA Extraction Kit, Cat. No. 10023), following the manufacturer's protocol for blood samples. After extraction, the DNA was quantified using NanoDrop Microvolume Spectrophotometers (Thermo Fisher, USA), ensuring accurate DNA concentration and purity measurement.

### 2.5. Identification of VAGs by Polymerase Chain Reaction (PCR)

The targeted genes were amplified using specific primers (listed in Table 2) with the PCR thermal cycler (T100TM, Bio-Rad, USA). The PCR protocol used for amplifying VAGs followed a precise thermal cycling process. A predenaturation step was initially performed at 94°C for 5 min to ensure complete DNA strand separation. This was followed by 35 amplification cycles, beginning with denaturation at 94°C for 30 s. For each cycle, the annealing temperatures varied according to the specific primers (Table 2), with an annealing duration of 30 s. The extension phase was carried out at 68°C for 1 min. After the cycles, a final elongation step at 72°C for 3 min was included to ensure the complete extension of the amplified DNA strands.

The final PCR product was stored at 4°C for subsequent use. The ingredients for the PCR mixture are as follows: 6.5 μL of nuclease-free water, 12.5 μL of mastermix (EmeraldAmp GT PCR Master Mix, Japan), 10 pmoles of forward and reverse primers (0.5 μL and 0.5 μL, respectively) (Microgen, Russia), and 5 μL of DNA template. The DNA template was finally added after properly mixing all components in the PCR tubes. After that, the thermocycler was heated to 94°C, and the PCR tubes were inserted. Eleven *E. coli* genes associated with virulence are amplified using conventional PCR: *omp*T, *hly*F, *iss*, *iut*A, *pap*C, *iuc*D, *tsh*, *irp*-2, *cva/cvi*, *ast*A, and *iro*N (Table 2) [10].

### 2.6. Agarose Gel Electrophoresis

A 1.5% gel was prepared by dissolving 0.6 g of agarose (CSL-AG100, Cleaver Scientific, USA) in 40 mL of 0.5 × TAE buffer (T4415, Sigma, USA) by heating the mixture in a microwave until the agarose was completely dissolved. Then, 3 μL of ethidium bromide was added as a DNA dye to visualize the DNA. Once the gel cooled to approximately 60°C, it was carefully poured into a dual comb caster and left undisturbed for 1 h. After solidification, the gel was placed in an electrophoresis tank (multiSUB Mini, Cleaver Scientific, USA), and the PCR products were loaded onto the gel for analysis. Before loading, a tracking dye was added to the PCR products. For each row of wells on the gel plate, 5 μL of the PCR products was mixed with 2 μL of loading dye, bromophenol blue, to facilitate the visualization of their movement through the agarose gel. Additionally, 1 μL of a DNA molecular weight marker (100-bp DNA Ladder, New England Biolabs) was included to compare the molecular weights of the PCR amplicons. The samples were electrophoresed simultaneously by applying a voltage of 100 V to the gel for 50 min. The integrity of the DNA was evaluated by visualizing it under UV light with a gel documentation system (Bio-Rad Gel Doc EZ Imager Documentation, Berkeley, California).

### 2.7. Data Analysis

Data entry and analysis were performed using Microsoft Excel 2019. To determine the prevalence rate for each category, the number of positive tests was divided by the total number of samples examined. Multidrug resistance (MDR) was defined as resistance to more than three antibiotic classes among all the antibiotics tested. The multiple antibiotic resistance (MAR) index was calculated using the formula: *a* divided by *b*, where “*a*” represents the number of antibiotics to which an isolate showed resistance and “*b*” indicates the total number of antibiotics used in AST. The cutoff values specified in the manufacturer's brochure (HiMedia, India) were applied in accordance with the guidelines established by the CLSI (2021) [32] to evaluate the patterns of AMR, including resistant, intermediate, and sensitivity. The ANOVA (one-way) test was performed using R software (Version 4.4.1; https://www.r-project.org/).

## 3. Results

### 3.1. Prevalence of APEC and EEC

A total of 155 samples were subjected to comprehensive microbiological and biochemical analyses, revealing that 61.29% (*n* = 95) tested positive for *E. coli* (Supporting Figure 2). Among these, 44.21% (*n* = 42) were identified as APEC, whereas the remaining 55.79% (*n* = 53) were classified as EEC. The anatomical distribution of APEC isolates showed variable prevalence, with 23.81% (*n* = 10) from the air sac, 28.57% (*n* = 12) from the spleen, 26.19% (*n* = 11) from the intestine, 11.91% (*n* = 5) from the lungs, and 9.52% (*n* = 4) from the liver. The EEC isolates were recovered from various nonanatomical sources, including feces (33.96%, *n* = 18), feed (15.09%, *n* = 8), water (32.07%, *n* = 17), and air (18.86%, *n* = 10).

### 3.2. The AMR Profiles of APEC and EEC

**Figure 2:**
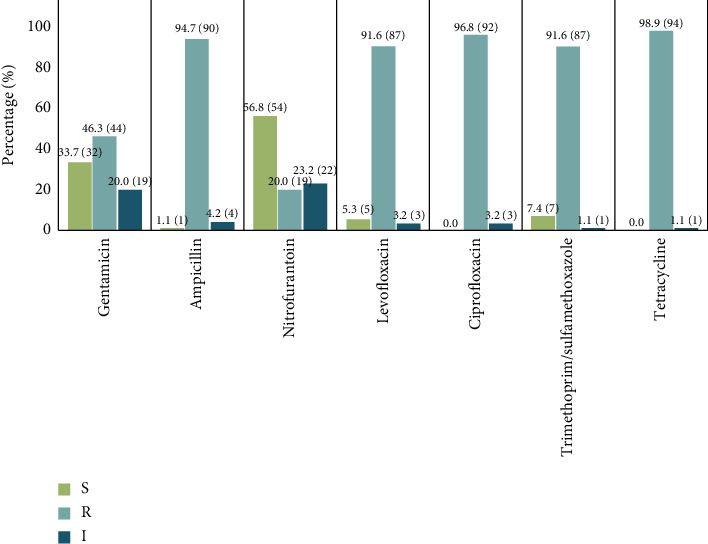
The AMR profile of the selected *E. coli* isolates based on the CLSI guideline (2021). The sensitive, resistant, and intermediate phenotypic characteristics are depicted by S, R, and I, respectively.

Based on the CLSI guidelines, tetracycline resistance was the most prevalent among the isolates, with 98.9% (*n* = 94) showing resistance. This was followed by high resistance rates to ciprofloxacin (96.8%, *n* = 92), ampicillin (94.7%, *n* = 90), levofloxacin (91.6%, *n* = 87), and the combination of trimethoprim and sulfamethoxazole (91.6%, *n* = 87). A notable percentage of isolates also exhibited resistance to gentamicin (46.3%, *n* = 44), whereas nitrofurantoin showed the lowest resistance rate at 20.0% (*n* = 19), making it comparatively less resistant among the antibiotics applied (Figure 2 and Supporting Figure 3).

**Figure 3:**
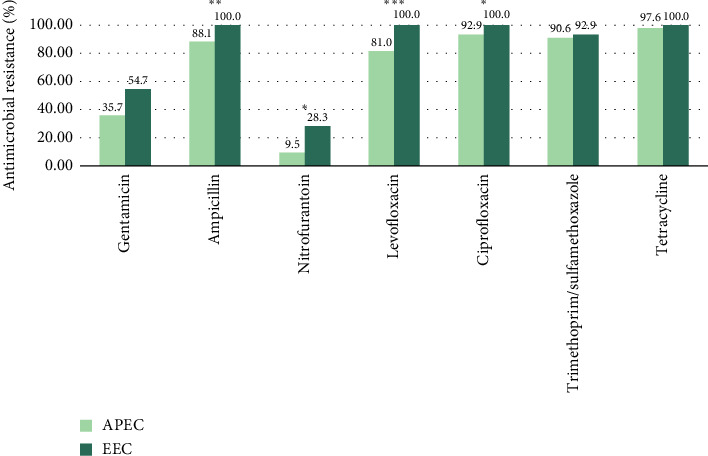
The AMR profile of the APEC and EEC isolates is depicted in light and dark green colors, respectively. *p* Value < 0.05 implies a statistically significant difference between the groups, where the number of asterisks (^∗^*p* < 0.05, ^∗∗^*p* < 0.01, and ^∗∗^*p* < 0.001) confers the magnitude of the differences.

The AMR profile of APEC and EEC isolates revealed significant differences across several antibiotics. Resistance to ampicillin was markedly higher among EEC isolates (100%, *n* = 53) compared to APEC (88.1%, *n* = 37), with a statistically significant difference (*p*=0.00951). Similarly, levofloxacin resistance was observed in all EEC isolates (100%, *n* = 53) but in 81.0% (*n* = 34) of APEC isolates, showing a highly significant difference (*p*=0.000731). The APEC isolates also exhibited lower resistance to ciprofloxacin (92.9%, *n* = 39) than EEC (100%, *n* = 53), with a statistically significant difference (*p*=0.0487). Notably, nitrofurantoin resistance was substantially lower in APEC (9.52%, *n* = 4) compared to EEC (28.3%, *n* = 15) isolates, with a significant difference (*p*=0.023). Although resistance to gentamicin was lower in APEC (35.7%, *n* = 15) than in EEC (54.7%, *n* = 29) isolates, the difference was not statistically significant (*p*=0.0662). Resistance to trimethoprim/sulfamethoxazole was notably high in both APEC (90.6%, *n* = 48) and EEC (92.9%, *n* = 39) isolates. Similarly, tetracycline resistance was detected in 97.6% (*n* = 41) of APEC isolates and 100% (*n* = 53) of EEC isolates. Despite the elevated resistance rates, statistical analysis revealed no significant differences between the two groups for either antibiotic (*p* > 0.05) (Figure 3, Table 3, and Supporting Table 2).

### 3.3. Prevalence of MDR and MAR Indices

The majority of the *E. coli* isolates exhibited the MDR phenotype, whereas 98.94% of the isolates were resistant to at least three classes of antibiotics. Notably, 10.52% of the isolates exhibited a resistance phenotype to all six classes of antibiotics. Furthermore, 40.0% of the isolates were resistant to five classes of antibiotics when tetracycline was included. Markedly, this resistance phenotype decreased to 8.42% when sulfonamide replaced tetracycline, suggesting a lower resistance rate to sulfonamide compared to tetracycline within this particular cohort (Table 4). When analyzing the resistance phenotype to four antibiotic classes, 40.0% of the isolates were found to be resistant to all antibiotics. However, the resistance rate increased slightly to 43.16% when tetracycline was assessed as the fourth antibiotic (replacing sulfonamide). An important finding emerged when resistance patterns were limited to four specific antibiotic groups: beta-lactam, quinolone, sulfonamide, and tetracycline. In these cases, the percentage of MDR isolates surged to between 80% and 85.26%, a substantial increase compared to the overall resistance rates (Table 4). Additionally, 43.16% of the isolates exhibited resistance to three groups of antibiotics when the combination included nitrofurantoin, gentamicin, beta-lactam, and tetracycline. However, the percentage of MDR isolates escalated to 80%–90% when the three antibiotic groups were confined to quinolone, sulfonamide, tetracycline, and beta-lactam, indicating a higher resistance trend with this specific grouping. Lastly, the resistance rate dropped significantly, falling below 15%, when the three antibiotic groups included gentamicin, nitrofurantoin, beta-lactam, and quinolone. However, 8.42% of the total isolates were found to be resistant to all seven antibiotics tested, indicating complete resistance and highlighting the severity of the issue (Table 4).

The study also calculated the MAR index for each isolate. Among the samples, 7.36% (*n* = 7) had a MAR index of 0.6, suggesting moderate antibiotic resistance. A significant proportion of the isolates, 43.15% (*n* = 41), showed a MAR index of 0.7, whereas 37.79% (*n* = 36) had a MAR index of 0.9. Notably, 8.42% (*n* = 8) of the isolates had a MAR index of 1.0, reflecting complete resistance to all tested antibiotic classes

### 3.4. Distribution of VAGs in APEC and EEC Isolates

**Figure 4:**
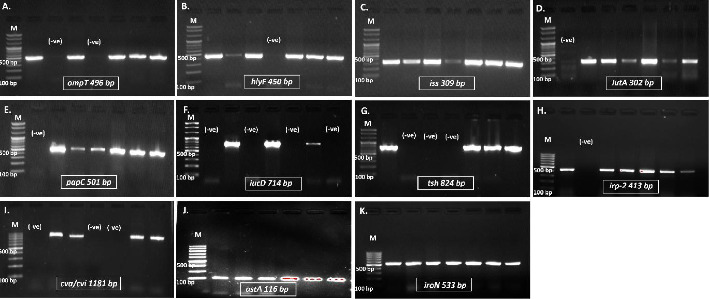
Visualization of VAGs from APEC and EEC isolates, including (a) *omp*T, (b) *hly*F, (c) *iss*, (d) *iut*A, (e) *pap*C, (f) *iuc*D, (g) *tsh*, (h) *irp*-2, (i) *cva/cvi*, (j) *ast*A, and (k) *iro*N. (*M* = 100 bp DNA ladder; (−ve) = absence of VAGs).

**Figure 5:**
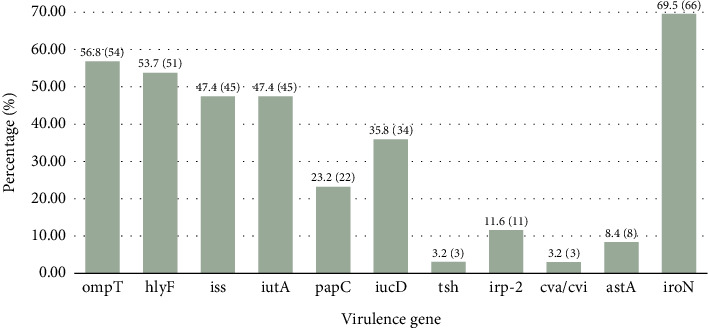
The prevalence of VAGs among the selected APEC and EEC isolates. The overall prevalence of VAGs is depicted in the bar chart.

Conventional PCR and gel electrophoresis were used to identify and visualize the presence of essential VAGs in *E. coli* isolates. This research focused on genes including *omp*T, *hly*F, *iss*, *iut*A, *pap*C, *iuc*D, *tsh*, *irp*-2, *cva/cvi*, *ast*A, and *iro*N among a cohort of 95 confirmed *E. coli* isolates (Figure 4 and Supporting File 1). The *iro*N was the most prevalent VAG among the isolates, comprising 69.5% (*n* = 66) of the total. This was followed by *omp*T (56.8%, *n* = 54), *hly*F (53.7%, *n* = 51), *iss* (47.4%, *n* = 45), and *iut*A (47.5%, *n* = 45). Significant quantities of other VAGs were identified, with *pap*C found in 23.2% (*n* = 22) of isolates and *iuc*D in 35.8% (*n* = 34). The *tsh* gene was identified in 3.2% (*n* = 3), but *irp*-2 was found in 11.6% (*n* = 11). Additionally, *cva/cvi* was identified in 3.2% (*n* = 3) of isolates, whereas *ast*A was detected in 8.4% (*n* = 8) (Figure 5).

**Figure 6:**
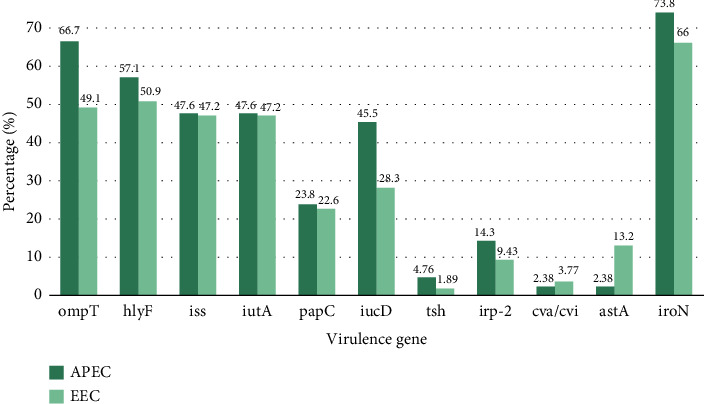
The prevalence of VAGs among the selected APEC and EEC isolates. The group-based prevalence of VAGs among APEC and EEC is depicted in dark and light green colors, respectively. *p* Value < 0.05 implies a statistically significant difference between the groups, where the number of asterisks (^∗^*p* < 0.05, ^∗∗^*p* < 0.01, and ^∗∗^*p* < 0.001) confers the magnitude of the differences.

Among the listed VAGs, *iro*N was the most prevalent VAG in both APEC (73.8%, *n* = 31) and EEC (66.0%, *n* = 35) isolates. This was followed by *omp*T, where APEC had 66.7% (*n* = 28) and EEC had 49.1% (*n* = 26). The *hly*F gene exhibited moderate prevalence in both groups, found in 57.1% (*n* = 24) of APEC isolates and 50.9% (*n* = 27) of EEC isolates. Despite the high prevalence, statistical analysis revealed no significant differences between the two groups for either VAGs (*p* > 0.05) (Figure 6, Table 5, and Supporting Table 3).

The *iss* and *iut*A genes exhibited similar prevalence rates in APEC (47.6%, *n* = 20) and EEC (47.2%, *n* = 25). Similarly, the *pap*C gene was detected in 23.8% (*n* = 10) of APEC and 22.6% (*n* = 12) of EEC isolates. The iron acquisition gene *iuc*D was more prevalent in APEC (45.5%, *n* = 19) than in EEC (28.3%, *n* = 15). Nonetheless, there were no significant differences observed between the two groups for the VAGs (*p* > 0.05). Other VAGs *tsh*, *irp*-2, *cva/cvi*, and *ast*A exhibited relatively low prevalence in both APEC and EEC isolates. In APEC, the prevalence of *tsh* was 4.76% (*n* = 2), *irp*-2 was 14.3% (*n* = 6), *cva/cvi* was 2.38% (*n* = 1), and *ast*A was also 2.38% (*n* = 1). In contrast, the EEC isolates showed slightly different distributions, with *tsh* identified in 1.89% (*n* = 1), *irp*-2 in 9.43% (*n* = 5), *cva/cvi* in 3.77% (*n* = 2), and *ast*A in 13.2% (*n* = 7) of the isolates. However, no significant changes were noted between the two groups for the VAGs (*p* > 0.05) (Figure 3, Table 3, and Supporting Table 3).

## 4. Discussion

This comprehensive evaluation of *E. coli* in broiler and environmental samples elucidates the intricate relationship between poultry health, AMR, and virulence factors. With 61.29% of the samples testing positive for *E. coli*, this high detection rate indicates significant contamination within the broiler environment, posing risks to poultry health and public health through the potential transfer of resistant strains. Notably, this prevalence of *E. coli* aligned with the previous study reported from Bangladesh [23, 25, 33]. The results showed a significant variation in *E. coli* strains, with 44.21% classified as APEC and 55.79% as EEC, emphasizing a substantial environmental reservoir. Prior research in Bangladesh found a much higher frequency of APEC compared to the current study [20–22, 24, 25]. Notably, the occurrence of APEC identified in this study contrasts with earlier findings from research conducted in other geographic areas, such as India [2, 34, 35], Nepal [7], and Pakistan [36]. The findings of this study indicate a higher prevalence than in certain studies conducted in India [35]; however, other Indian studies have reported even higher prevalence rates than those identified in the current study [2, 34]. Studies conducted in Nepal and Pakistan have also reported higher prevalence rates of APEC compared to the findings of the present study [7, 36].

The distribution of APEC isolates across various anatomical sites revealed a systemic spread, with significant numbers coming from the spleen (28.57%), intestine (26.19%), and air sac (23.81%). This suggests that these organs are primary targets during infection. The reduced recovery levels from the lungs (11.91%) and liver (9.52%) could indicate either waned colonization or restricted systemic spread in particular cases. Conversely, the EEC isolates predominantly originated from feces (33.96%) and water (32.07%), highlighting the significance of fecal contamination and water quality as vital environmental sources. The detection of EEC in the air (18.86%) and feed (15.09%) highlights the critical need for robust biosecurity and hygiene practices in poultry settings. In this study, all *E. coli* isolates showed varying levels of resistance to key antimicrobials, including gentamicin, ampicillin, nitrofurantoin, levofloxacin, ciprofloxacin, sulfamethoxazole/trimethoprim, and tetracycline. The high rate of tetracycline resistance (98.9%) among the isolates is alarming, as tetracycline is commonly used in poultry medicine. This high resistance rate of tetracycline was also reported in different studies from Bangladesh [20, 24, 25, 37, 38]. Similarly, the substantial resistance to ciprofloxacin (96.8%), ampicillin (94.7%), and levofloxacin (91.6%) suggested that these antibiotics, which are critical for both human and poultry treatments, may be losing their efficacy against *E. coli* infections. This high resistance rate was also aligned with the other studies in Bangladesh [20, 21, 24, 25, 37, 38]. The AMR profile revealed notable differences between APEC and EEC isolates. The isolates from EEC demonstrated consistently elevated resistance levels, especially against ampicillin, levofloxacin, and ciprofloxacin, with statistically significant differences (*p* < 0.05). This suggests a potential overexposure to antimicrobials in the environment or an increased acquisition of resistance determinants. Levofloxacin resistance was found to be universal in EEC at 100%, whereas it was lower in APEC at 81.0%. This indicates a troubling trend of fluoroquinolone resistance in environmental strains. Although the resistance to nitrofurantoin was typically low, it was notably higher in EEC at 28.3% compared to APEC, which stood at 9.52%. The level of gentamicin resistance was elevated in EEC, albeit without statistical significance, suggesting a potential emerging trend. Significant resistance rates to trimethoprim/sulfamethoxazole and tetracycline in both groups (> 90%) indicate a prevalent issue, probably stemming from their extensive application in poultry. However, the AMR profile of APEC isolates identified in this study aligns with findings from previous studies conducted in India [35], Nepal [10], and Pakistan [14].

One of the most concerning observations was the extensive MDR exhibited by the isolates. In this study, 98.94% of *E. coli* exhibited resistance to at least three antibiotic classes. This study also demonstrated alarming levels of antibiotic resistance among isolates, with 10.52% showing resistance to all six tested antibiotic classes. Additionally, 40.0% of *E. coli* were resistant to five classes when tetracycline was included, but this percentage dropped to 8.42% when sulfonamide replaced tetracycline. Conversely, 40.0% of the *E. coli* were resistant to four antibiotic classes when sulfonamide was included, rising to 43.16% when tetracycline replaced sulfonamide. Importantly, MDR *E. coli* isolates increased significantly (80%–85.26%) when four groups—beta-lactam, quinolone, sulfonamide, and tetracycline—were tested. Moreover, 43.16% of the isolates showed resistance to three antibiotic groups, including nitrofurantoin, gentamicin, beta-lactam, and tetracycline, with MDR rates rising to 80%–90% when quinolone, sulfonamide, tetracycline, and beta-lactam were tested together. Notably, the MDR percentage dropped below 15% when the antibiotic groups included gentamicin, nitrofurantoin, beta-lactam, and quinolone, highlighting variability in resistance patterns based on antibiotic combinations. These findings emphasize the widespread misuse of antibiotics within Bangladesh's poultry sector. Importantly, the transmission of this resistance phenotype occurs through mechanisms such as conjugation, transformation, and transduction. Furthermore, the resistance phenotype is also associated with several biological processes, including bacterial growth in the stationary phase, biofilm formation, and persistence [24, 39].

The transmission of AMR to humans primarily occurs through the food chain, often by consuming improperly cooked meat or through unsafe handling during processing [40]. Additionally, poultry waste plays a significant role in disseminating resistance into the environment [41]. However, raising farmers' awareness about the proper use of antimicrobials has improved AMR conditions within the farming environment [42]. The MAR index values, which reflect the level of antibiotic resistance, also provide a comprehensive profile of AMR isolates. With 43.15% of the *E. coli* isolates having a MAR index of 0.7% and 37.79% with an index of 0.9, it is evident that most isolates possess a high degree of resistance to multiple antibiotics. The 8.42% of isolates with a MAR index of 1.0, representing complete resistance to all tested antibiotics, emphasize the urgency of addressing this growing threat through better antimicrobial stewardship and the development of alternative treatments.

In addition to the AMR findings, the detection of multiple VAGs among the *E. coli* isolates provides insight into the potential pathogenicity of these strains. The high prevalence of the iroN gene (69.5%) indicates a strong iron acquisition system, which is essential for bacterial survival in host environments. Similarly, the presence of *omp*T (58.8%) and *hly*F (53.7%) suggested that these isolates possess enhanced mechanisms for evasion of host immune responses and tissue invasion. Nearly half of the isolates carried the *iss* (47.4%) and *iut*A (47.5%) genes, which highlight the potential for these strains to evade host immune defenses and acquire iron, further contributing to their pathogenic potential. The identification of less common virulence genes such as *iuc*D (35.8%), *pap*C (23.2%), *irp*-2 (11.6%), and *ast*A (8.4%) suggested that some isolates may possess additional traits which enhance their ability to colonize and cause disease in poultry. Previous studies in Bangladesh also revealed the presence of such VAGs in APEC isolates [24, 38]. Although the prevalence of some virulence factors was relatively low, such as *cva/cvi* (3.2%) and *tsh* (3.2%), their presence in a subset of isolates highlights the genetic diversity within *E. coli* strains. However, the prevalence of the *cva/cvi* gene observed in the present study is fully consistent with that reported in previous studies conducted in Bangladesh [24]. A related study in Nepal identified these genes in APEC isolates, demonstrating similar frequency trends [10]. Nonetheless, a separate investigation conducted in Pakistan found that the *iss*, *tsh*, *iro*N, and *iut*A genes were present in APEC isolates [14].

The VAG profiling of APEC and EEC isolates demonstrated significant genetic similarities, especially with high-prevalence genes such as *iro*N, *omp*T, and *hly*F. *iro*N, an iron acquisition gene vital for bacterial survival in iron-deficient settings such as the host, was the most abundant VAG in both APEC (73.8%) and EEC (66.0%), indicating its key contribution to both pathogenicity and environmental endurance. Likewise, *omp*T and *hly*F exhibited moderate to high incidence in both cohorts, substantiating the concept that ambient *E. coli* may include characteristics often linked to virulence. Genes such as *iss*, *iut*A, *pap*C, and *iuc*D exhibited widespread distribution, with no statistically significant differences, suggesting that these virulence factors are not limited to APEC. The lower-prevalence VAGs (*tsh*, *irp*-2, *cva/cvi*, and *ast*A) exhibited variable distribution however remained rare in both groups. The existence of virulence genes in environmental isolates raises concerns about their ability to serve as reservoirs for gene exchange and adaptation [43]. The environmental reservoirs of *E. coli*, which harbor virulence determinants, have the potential to promote the horizontal transfer of these genes to other species, including those associated with humans and livestock [44]. Selective pressures, including the extensive use of antibiotics in agriculture and the presence of susceptible hosts, could further hasten the exchange of genes. Therefore, environmental isolates function not just as passive carriers but also as active contributors to the development of pathogenicity and antibiotic resistance [45]. To deal with this risk, integrated surveillance and management techniques involving agricultural practices, environmental monitoring, and public health interventions must be implemented, in compliance with the One Health approach of coordinated action across ecological and health domains [45–47]. Overall, the combination of widespread MDR and multiple virulence genes suggested that these *E. coli* isolates pose a significant threat to poultry and humans through potential zoonotic transmission. These findings emphasize the need for ongoing surveillance, better biosecurity practices in poultry farming, and more judicious use of antibiotics to curb the spread of resistant and virulent bacterial strains.

## 5. Conclusion

This comprehensive research uncovers a hindering correlation among antibiotic resistance, virulence, and environmental survival of *E. coli* in broiler environments. The elevated rate of *E. coli*, particularly APEC and EEC strains, along with extensive antibiotic resistance and various VAGs, highlights the considerable risk these bacteria pose to poultry health, environmental integrity, and public health. The heightened resistance to key antibiotics, particularly tetracycline and fluoroquinolones, emphasizes the urgent need for antimicrobial management. The presence of virulence genes in clinical and environmental isolates underscores the potential for gene transfer and zoonotic risk. These results need stringent monitoring, enhanced farm hygiene and biosecurity measures, and restricted antibiotic use to address the increasing likelihood of resistant and harmful *E. coli* strains in chicken production systems.

## Figures and Tables

**Table 1:** Antibiotics used for antibiotic susceptibility test (AST) of APEC and EEC and their resistance breakpoints according to the CLSI guideline 2021.

			Zone of inhibition in diameter (mm)		
Antibiotic classes	Name of the antibiotics	Disk potency (mcg)	Resistant (R)	Intermediate (I)	Susceptible (S)
Aminoglycosides	Gentamicin (CN 10)	10	≤ 12	13–14	≥ 15

β-Lactam	Ampicillin (AMP 10)	10	≤ 13	14–16	≥ 17

Nitrofurantoin	Nitrofurantoin (F 300)	300	≤ 14	15–16	≥ 17

Quinolone	Levofloxacin (LEV 5)	5	≤ 16	17–20	≥ 21
	Ciprofloxacin (CIP 5)	5	≤ 20	21–30	≥ 31

Sulfonamide	Trimethoprim and sulfamethoxazole (SXT 25)	25	≤ 10	11–15	≥ 16

Tetracyclines	Tetracyclines (TE 30)	30	≤ 11	12–14	≥ 15

**Table 2:** The list of common VAGs of APEC and EEC with their annealing temperature and product size.

Virulent-associated gene (VAG)	Primer sequence (5′–3′)	Annealing temperature (°C)	Size (bp)
*iutA*	F: GGCTGGACATCATGGGAACTGG	66	302
	R: CGTCGGGAACGGGTAGAATCG		

*iss*	F: CAGCAACCCGAACCACTTGATG	66	309
	R: AGCATTGCCAGAGCGGCAGAA		

*papC*	F: TGATATCACGCAGTCAGTAGC	55	501
	R: CCGGCCATATTCACATAA		

*iucD*	F: ACAAAAAGTTCTATCGCTTCC	55	714
	R: CCTGATCCAGATGATGCTC		

*tsh*	F: ACTATTCTCTGCAGGAAGTC	56	824
	R: CTTCCGATGTTCTGAACGT		

*irp-2*	F: AAGGATTCGCTGTTACCGGAC	61	413
	R: AACTCCTGATACAGGTGGC		

*ompT*	F: TCATCCCGGAAGCCTCCCTCACTACTAT	65	496
	R: TAGCGTTTGCTGCACTGGCTTCTGATAC		

*hlyF*	F: GGCCACAGTCGTTTAGGGTGCTTACC	65	450
	R: GGCGGTTTAGGCATTCCGATACTCAG		

*iroN*	F: AATCCGGCAAAGAGACGAACCGCCT	68	533
	R: GTTCGGGCAACCCCTGCTTTGACTTT		

*cva/cvi*	F: TGGTAGAATGTGCCAGAGCAAG	61	1181
	R: GAGCTGTTTGTAGCGAAGCC		

*astA*	F: TGCCATCAACACAGTATATCC	68	116
	R: TCAGGTCGCGAGTGACGGC		

**Table 3:** The antimicrobial resistance profile of the APEC and EEC isolates.

Antibiotics	APEC % (*n*)					EEC % (*n*)				*p* Value
	Air sac	Spleen	Liver	Intestine	Lungs	Water	Feed	Feces	Air	
Gentamicin	30 (3)	66.66 (8)	—	27.27 (3)	20 (1)	41.17 (7)	50 (4)	61.11 (11)	70 (7)	0.0662
Ampicillin	100 (10)	75 (9)	—	90.90 (10)	80 (4)	100 (17)	100 (8)	100 (18)	100 (10)	0.00951^∗∗^
Nitrofurantoin	—	16.66 (2)	—	—	40 (2)	29.41 (5)	25 (2)	33.33 (2)	20 (2)	0.023^∗^
Levofloxacin	80 (8)	91.66 (11)	100 (4)	81.81 (9)	40 (2)	100 (17)	100 (8)	100 (18)	100 (10)	0.000731^∗∗∗^
Ciprofloxacin	90 (9)	100 (12)	100 (4)	90.90 (10)	80 (4)	100 (17)	100 (8)	100 (18)	100 (10)	0.0487^∗^
Trimethoprim/sulfamethoxazole	100 (10)	100 (12)	100 (4)	72.72 (8)	100 (5)	94.11 (16)	87.5 (7)	88.88 (16)	90 (9)	0.693
Tetracycline	90 (9)	100 (12)	100 (4)	100 (11)	100 (5)	100 (17)	100 (8)	100 (18)	100 (10)	0.264

*Note:* The asterisks (^∗^*p* < 0.05, ^∗∗^*p* < 0.01, and ^∗∗∗^*p* < 0.001) confer the magnitude of the differences, where *p* value < 0.05 implies a statistically significant difference.

**Table 4:** The prevalence of multidrug resistance (MDR) and multiple antibiotic resistance (MAR) among the APEC and EEC isolates.

Number of antibiotic classes	Resistance type	Resistant pattern	No. of isolates	Percentage	MAR index
6	MDR	CN (10) + AMP (10) + F (300) + LEV (5) + SXT (25) + TE (30)	7	10.52	0.86
		CN (10) + AMP (10) + F (300) + CIP (5) + SXT (25) + TE (30)	3		

5	MDR	CN (10) + AMP (10) + LEV (5) + SXT (25) + TE (30)	38	40.0	0.71
		CN (10) + AMP (10) + CIP (5) + SXT (25) + TE (3)	38		
		CN (10) + AMP (10) + LEV (5) + SXT (25) + F (300)	8	8.42	
		CN (10) + AMP (10) + CIP (5) + SXT (25) + F (300)	8		

4	MDR	CN (10) + AMP (10) + LEV (5) + SXT (25)	38	40.0	0.57
		CN (10) + AMP (10) + CIP (5) + SXT (25)	38		
		CN (10) + AMP (10) + LEV (5) + TE (30)	41	43.16	
		CN (10) + AMP (10) + CIP (5) + TE (30)	41		
		CN (10) + LEV (5) + SXT (25) + TE (30)	41		
		CN (10) + CIP (5) + SXT (25) + TE (30)	41		
		AMP (10) + CIP (5) + SXT (25) + TE (30)	81	85.26	
		AMP (10) + F (300) + SXT (25) + TE (30)	11	11.58	
		AMP (10) + LEV (5) + SXT (25) + TE (30)	76	80	

3	MDR	CN (10) + AMP (10) + LEV (5)	41	43.16	0.43
		CN (10) + AMP (10) + CIP (5)	41		
		LEV (5) + SXT (25) + TE (30)	80	84.21	
		CIP (5) + SXT (25) + TE (30)	85	89.47	
		CN (10) + SXT (25) + TE (30)	41	43.16	
		AMP (10) + LEV (5) + SXT (25)	76	80	
		AMP (10) + CIP (5) + SXT (25)	81	85.26	
		F (300) + CIP (5) + SXT (25)	14	14.74	
		F (300) + LEV (5) + SXT (25)	13	13.68	
		CN (10) + AMP (10) + F (300)	10	10.52	

**Table 5:** The prevalence of VAGs among the APEC and EEC isolates.

Virulent-associated gene (VAG)	APEC % (*n*)					EEC % (*n*)				*p* Value
	Air sac	Spleen	Liver	Intestine	Lungs	Water	Feed	Feces	Air	
*ompT*	70 (7)	83.33 (10)	50 (2)	63.63 (7)	40 (2)	47.05 (8)	62.5 (5)	50 (9)	40 (4)	0.0869
*hlyF*	60 (6)	66.66 (8)	50 (2)	54.54 (6)	40 (2)	47.05 (8)	62.5 (5)	55.55 (10)	40 (4)	0.552
*iss*	50 (5)	83.33 (10)	25 (1)	27.27 (3)	20 (1)	47.05 (8)	62.5 (5)	44.44 (8)	40 (4)	0.966
*iutA*	10 (1)	83.33 (10)	50 (2)	45.45 (5)	40 (2)	52.94 (9)	62.5 (5)	44.44 (8)	30 (3)	0.966
*papC*	30 (3)	50 (6)	—	9.09 (1)	—	23.52 (4)	25 (2)	27.77 (5)	10 (1)	0.895
*iucD*	40 (4)	50 (6)	50 (2)	45.45 (5)	40 (2)	29.41 (5)	25 (2)	38.88 (7)	10 (1)	0.089
*tsh*	—	8.33 (1)	—	—	—	—	—	5.55 (1)	—	0.432
*irp-2*	—	16.66 (2)	25 (1)	27.27 (3)	—	—	12.5 (1)	16.66 (3)	10 (1)	0.468
*cva/cvi*	—	8.33 (1)	—	—	—	—	—	5.55 (1)	10 (1)	0.704
*astA*	—	8.33 (1)	—	—	—	11.76 (2)	37.5 (3)	5.55 (1)	10 (1)	0.0601
*iroN*	80 (8)	75 (9)	50 (2)	90.90 (10)	40 (2)	64.70 (11)	62.5 (5)	55.55 (10)	90 (9)	0.419

## Data Availability

The data that support the findings of this study are available in the supporting information of this article.

## Supporting Information

Supporting figures.

Supporting Information 1 Supporting Figure 1: Samples were collected from key anatomical sites of deceased broilers, including the liver, lung, spleen, intestine, air sac, and feces, as well as environmental samples from farms, such as air, feed, and water.

Supporting Information 2 Supporting Figure 2: Confirmatory microbiological, microscopical, and biochemical tests. The APEC and EEC isolates were initially confirmed by the laboratory culturing in the selective MacConkey (A) and EMB agar media (B), which were further confirmed by Gram staining and direct microscopy (C). The biochemical tests, including TSI (D), MIU (E), and Simmons citrate (F) test, were then used to confirm the E. coli isolates.

Supporting Information 3 Supporting Figure 3: Phenotypic AMR profile of the APEC and EEC isolates. The Kirby–Bauer disk diffusion method demonstrates resistance phenotypes of ampicillin (A), levofloxacin (B), trimethoprim and sulfamethoxazole (C), tetracycline (D), gentamicin (E), ciprofloxacin (F), and nitrofurantoin (G).

Supporting Information 4 Supporting Table 1: Biochemical characterization of APEC.

Supporting Information 5 Supporting Table 2: The antimicrobial profile of APEC and EEC isolates.

Supporting Information 6 Supporting Table 3: The prevalence of VAGs among the APEC and EEC isolates.

Supporting Information 7 Supporting File 1: Virulence-associated genes (VAGs) and antimicrobial resistance profile (phenotype).
