# Supplementary figures and images for: Multidrug Resistance and Virulence Gene Profiles of E. coli in Broiler Chickens: A Study From Noakhali, Bangladesh
Source: Vet Med Int. 2025 Nov 25;2025:1157843. doi: 10.1155/vmi/1157843 (PMC12672072; doi:10.1155/vmi/1157843)

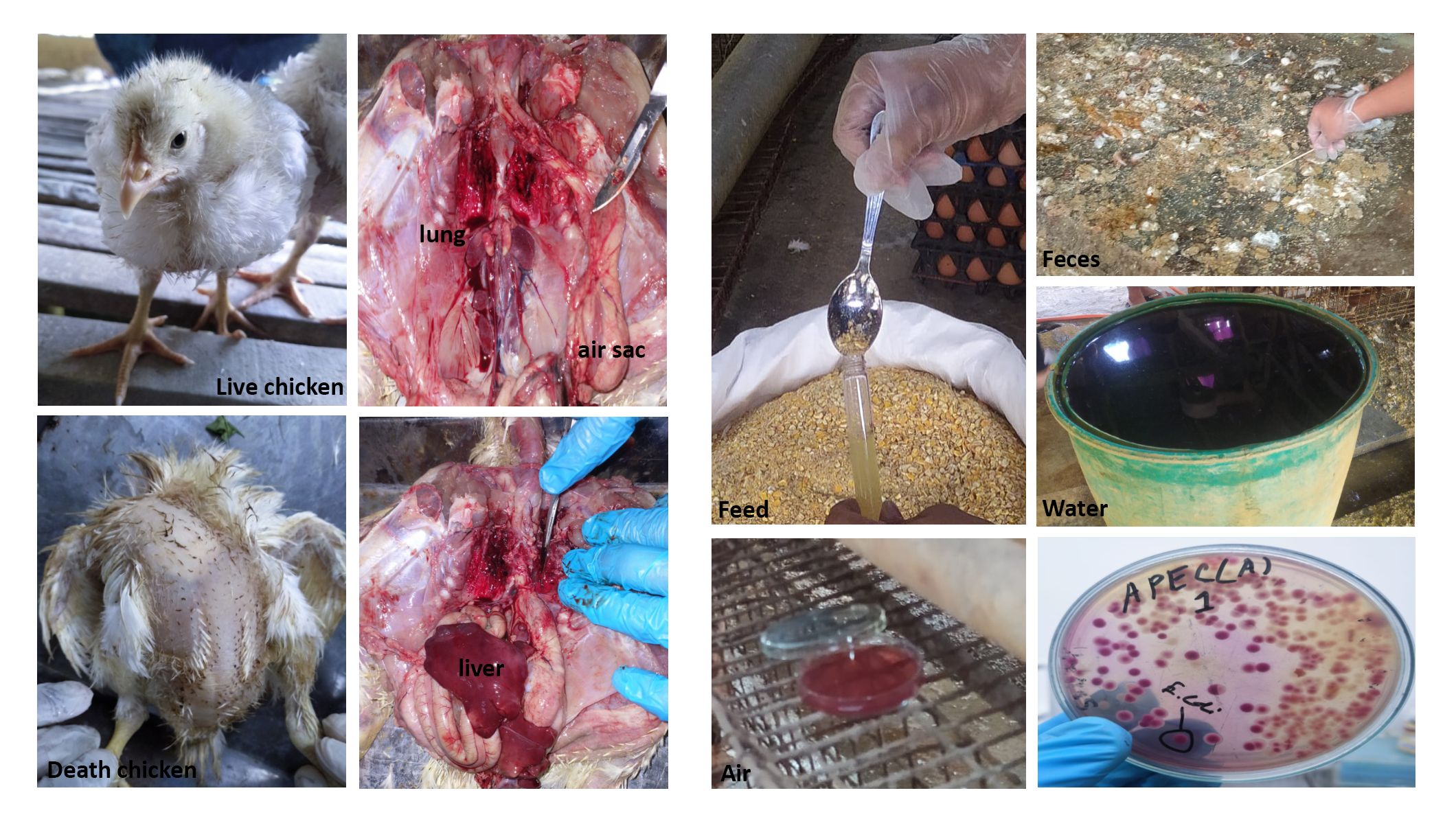

Supplement: Supporting Information 1 — Supporting Figure 1: Samples were collected from key anatomical sites of deceased broilers, including the liver, lung, spleen, intestine, air sac, and feces, as well as environmental samples from farms, such as air, feed, and water. [file 1157843.f1.tif]

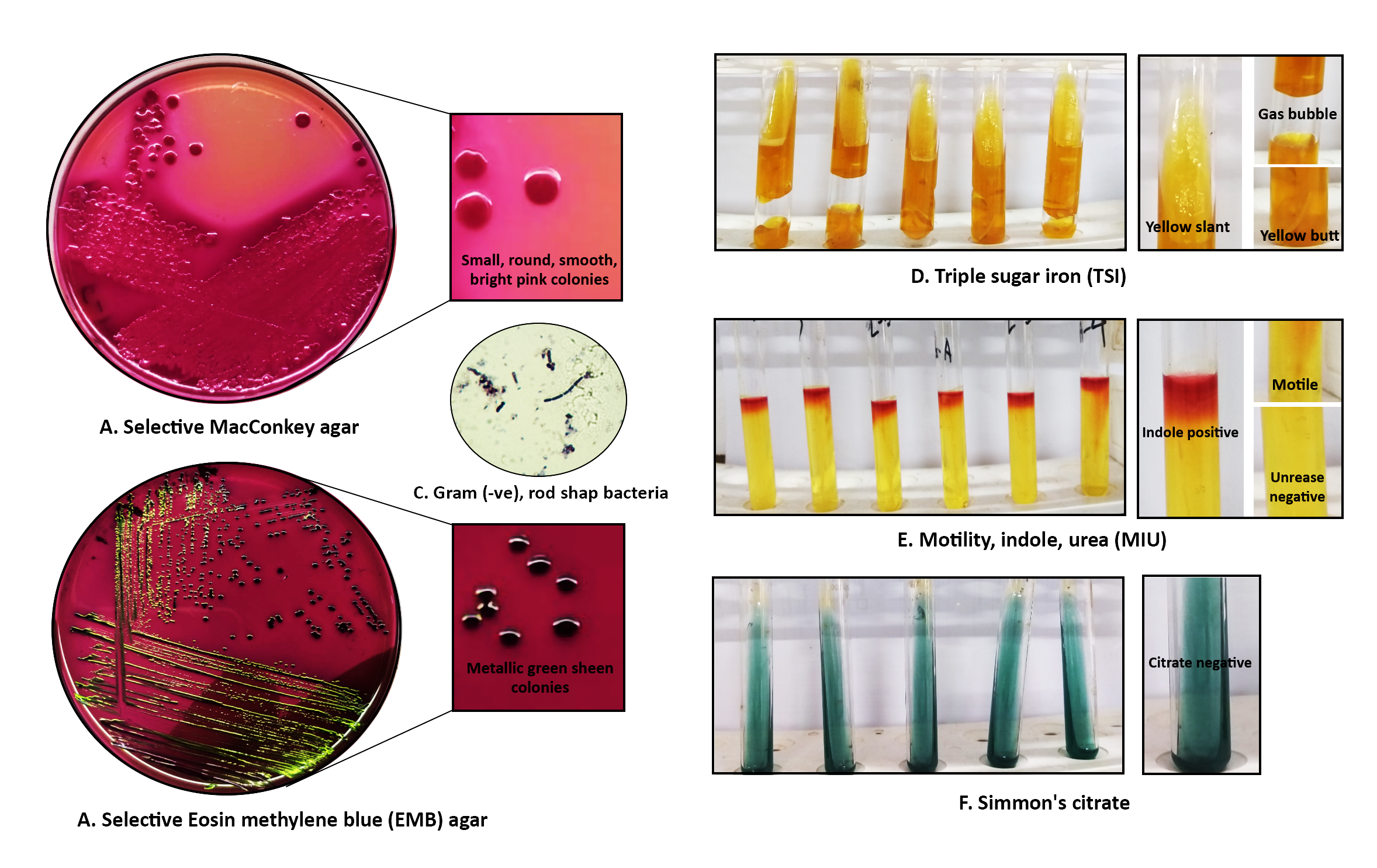

Supplement: Supporting Information 2 — Supporting Figure 2: Confirmatory microbiological, microscopical, and biochemical tests. The APEC and EEC isolates were initially confirmed by the laboratory culturing in the selective MacConkey (A) and EMB agar media (B), which were further confirmed by Gram staining and direct microscopy (C). The biochemical tests, including TSI (D), MIU (E), and Simmons citrate (F) test, were then used to confirm the E. coli isolates. [file 1157843.f2.tif]

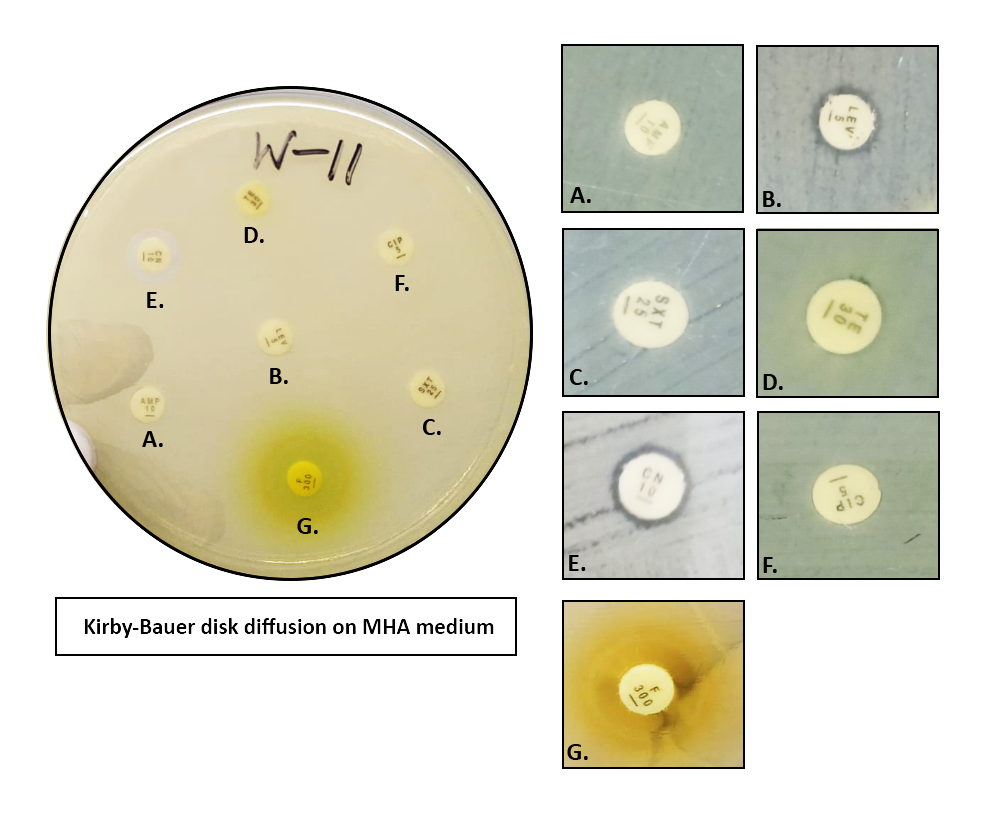

Supplement: Supporting Information 3 — Supporting Figure 3: Phenotypic AMR profile of the APEC and EEC isolates. The Kirby–Bauer disk diffusion method demonstrates resistance phenotypes of ampicillin (A), levofloxacin (B), trimethoprim and sulfamethoxazole (C), tetracycline (D), gentamicin (E), ciprofloxacin (F), and nitrofurantoin (G). [file 1157843.f3.tif]
