# Supplementary material for: Multidrug Resistance and Virulence Gene Profiles of E. coli in Broiler Chickens: A Study From Noakhali, Bangladesh
Source: Vet Med Int. 2025 Nov 25;2025:1157843. doi: 10.1155/vmi/1157843 (PMC12672072; doi:10.1155/vmi/1157843)
Supplement: Supporting Information 4 — Supporting Table 1: Biochemical characterization of APEC. [file 1157843.f4.docx]

| **Test** | **Feature** | **Remark** | |
| --- | --- | --- | --- |
|  |  | **APEC Positive** | **APEC Negative** |
| Triple sugar iron (TSI) agar | Slant | Yellow | Red |
|  | Butt | Yellow | Red |
|  | Gas | Yes | No |
|  | H2S | No | Yes (blackening) |
| MIU | Indole | Yes (Red color at surface) | No (Yellow color at surface) |
|  | Motility | Yes | No |
|  | Urease | No (Yellow color appearance) | Yes (pink color appearance) |
| Simmon's citrate | Citrate | No (blue color) | Yes (Green color) |

**Supplementary table 1.** Biochemical characterization of APEC.
