# Supplementary material for: Multidrug Resistance and Virulence Gene Profiles of E. coli in Broiler Chickens: A Study From Noakhali, Bangladesh
Source: Vet Med Int. 2025 Nov 25;2025:1157843. doi: 10.1155/vmi/1157843 (PMC12672072; doi:10.1155/vmi/1157843)
Supplement: Supporting Information 5 — Supporting Table 2: The antimicrobial profile of APEC and EEC isolates. [file 1157843.f5.docx]

**Supplementary table 2.** The antimicrobial profile of APEC and EEC isolates.

| **Antibiotics** | **APEC (n)** | **EEC (n)** | **P-value** |
| --- | --- | --- | --- |
| Gentamicin | 35.7 (15) | 54.7 (29) | 0.0662 |
| Ampicillin | 88.1 (37) | 100 (53) | 0.00951** |
| Nitrofurantoin | 9.52 (4) | 28.3 (15) | 0.023* |
| Levofloxacin | 81.0 (34) | 100 (53) | 0.000731*** |
| Ciprofloxacin | 92.9 (39) | 100 (53) | 0.0487* |
| Trimethoprim/Sulfamethoxazole | 90.6 (48) | 92.9 (39) | 0.693 |
| Tetracycline | 97.6 (41) | 100 (53) | 0.264 |
