# Supplementary material for: Multidrug Resistance and Virulence Gene Profiles of E. coli in Broiler Chickens: A Study From Noakhali, Bangladesh
Source: Vet Med Int. 2025 Nov 25;2025:1157843. doi: 10.1155/vmi/1157843 (PMC12672072; doi:10.1155/vmi/1157843)
Supplement: Supporting Information 6 — Supporting Table 3: The prevalence of VAGs among the APEC and EEC isolates. [file 1157843.f6.docx]

**Supplementary table 3.** The prevalence of VAGs among the APEC and EEC isolates.

| **Antibiotics** | **APEC** | **EEC** | **P-value** |
| --- | --- | --- | --- |
| ompT | 66.7 (28) | 49.1 (26) | 0.0869 |
| hlyF | 57.1 (24) | 50.9 (27) | 0.552 |
| iss | 47.6 (20) | 47.2 (25) | 0.966 |
| iutA | 47.6 (20) | 47.2 (25) | 0.966 |
| papC | 23.8 (10) | 22.6 (12) | 0.895 |
| iucD | 45.5 (19) | 28.3 (15) | 0.089 |
| tsh | 4.76 (2) | 1.89 (1) | 0.432 |
| irp-2 | 14.3 (6) | 9.43 (5) | 0.468 |
| cva/cvi | 2.38 (1) | 3.77 (2) | 0.704 |
| astA | 2.38 (1) | 13.2 (7) | 0.0601 |
| iroN | 73.8 (31) | 66.0 (35) | 0.419 |
